# Presumed LRP1-targeting transport peptide delivers β-secretase inhibitor to neurons in vitro with limited efficiency

**DOI:** 10.1038/srep34297

**Published:** 2016-09-29

**Authors:** Jong Ah Kim, Tommaso Casalini, Davide Brambilla, Jean-Christophe Leroux

**Affiliations:** 1 Institute of Pharmaceutical Sciences, Department of Chemistry and Applied Biosciences, ETH Zurich, Zurich, Switzerland.; 2 Institute of Chemical and Bioengineering, Department of Chemistry and Applied Biosciences, ETH Zurich, Zurich, Switzerland.

## Abstract

Interfering with the activity of β-secretase to reduce the production of Aβ peptides is a conceivable therapeutic strategy for Alzheimer’s disease. However, the development of efficient yet safe inhibitors is hampered by secondary effects, usually linked to the indiscriminate inhibition of other substrates’ processing by the targeted enzyme. Based on the spatial compartmentalization of the cleavage of the amyloid precursor protein by β-secretase, we hypothesized that by exploiting the endocytosis receptor low-density lipoprotein receptor-related protein it would be possible to direct an otherwise cell-impermeable inhibitor to the endosomes of neurons, boosting the drug’s efficacy and importantly, sparing the off-target effects. We used the transport peptide Angiopep to build an endocytosis-competent conjugate and found that although the peptide facilitated the inhibitor’s internalization into neurons and delivered it to the endosomes, the delivery was not efficient enough to potently reduce β-secretase activity at the cellular level. This is likely connected to the finding that in the cell lines we used, Angiopep’s internalization was not mediated by its presumed receptor to a significant extent. Additionally, Angiopep exploited different internalization mechanisms when applied alone or when conjugated to the inhibitor, highlighting the impact that drug conjugation can have on transport peptides.

Alzheimer’s disease (AD) is the most common type of dementia worldwide and is currently the sixth leading cause of death. The complex biology and long pre-symptomatic incubation period of the disease have greatly complicated the development of disease-modifying treatments and to date only palliative treatments with marginal benefits exist. While AD’s etiology continues to be the subject of active research, the neurotoxic aggregates of amyloid β-peptide (Aβ) have remained a central target of therapeutic strategies^1^,^2^,^3^. Their production and clearance from the brain have been studied exhaustively and accordingly, various attempts to modulate the balance between the two processes have been described^4^,^5^. Interfering with the activity of the Aβ-producing enzymes has yielded encouraging results and has validated the reduction of Aβ peptides as a therapeutic approach^4^. However, unacceptable side effects have often resulted in clinical trials being halted early on and so far no drugs have reached the market^6^.

Aβ peptides derive from the proteolytic cleavage of the amyloid precursor protein (APP), a constitutively expressed transmembrane glycoprotein, by the sequential actions of β- and γ-secretases. The sequence of these 37–42 amino acids peptides is rich in hydrophobic residues, making it prone to form self-aggregates associated with synaptic toxicity and neurodegeneration^1^,^7^,^8^. Given the numerous substrates of γ-secretase and the deleterious consequences of its pharmacological inhibition, β-secretase is considered a more suitable target to interfere with the amyloidogenic processing pathway of APP^6^,^9^. The β-secretase enzyme, also known as β-site amyloid precursor protein-cleaving enzyme 1 (BACE1), is a membrane-tethered aspartyl protease found at the surface of cells and the endocytic and secretory compartments^10^. Because its APP-cleaving activity mostly takes place in the endosomes where the acidic pH activates the enzyme, the internalization and subcellular processing of inhibitory drugs need to be considered in the design of therapeutic strategies^10^,^11^. Based on the proposed association of BACE1 to the cholesterol-rich lipid rafts of the plasma membrane^11^,^12^,^13^, a pioneering study coupled a sterol moiety to an inhibitory molecule to target it to the proximity of the enzyme and render it endocytosis-competent^14^. The conjugate was successfully processed by cells and inhibited BACE1 *in vivo*, showing the enzyme’s trafficking could be exploited for therapeutic purposes. Although in this case the administration route used was stereotaxic injection, which is not clinically translatable, the promising results obtained suggested that modifying an inhibitor molecule to target it for endocytosis constituted a valid approach to promote its cellular activity. This prompted us to explore other strategies that could not only boost the intracellular dose achieved, but also do so in a compartmentalized fashion (i.e. specifically in the endosomes), so that the side effects associated with unspecific inhibition of the cleavage of other BACE1’s substrates elsewhere in the cell would be reduced. Finally, an approach that could additionally overcome the blood-brain barrier (BBB) would be highly desirable as its intravenous administration would spare the need to deliver the drug through invasive procedures. The low-density lipoprotein receptor-related protein (LRP1) modulates the processing of APP by associating with it at the cell surface and directing it to the intracellular compartment inside of endosomal vesicles^15^,^16^. The LRP1 receptor is a multifunctional endocytosis receptor implicated in numerous biological processes including transcytosis of metabolites (such as Aβ) across the BBB^17^,^18^, and therefore constitutes a highly appealing target for the delivery of drugs to the central nervous system^19^. Similar to what has been described for BACE1, the presence of LRP1 is enriched at the cell’s surface lipid rafts. Not surprisingly, the receptor has been shown to interact with the secretase through its light chain and to be processed by the enzyme^20^. We hypothesized that designing a secretase inhibitory complex, which exploits the tripartite interaction between the LRP1 receptor, BACE1 and APP to block the proteolytic cleavage of the latter in a spatially confined fashion, could be advantageous both from an efficacy and specificity point of view. Firstly, promoting the endocytosis of the secretase inhibitor would enable higher concentrations of the drug to be achieved inside the endosomes, where the relevant enzymatic activity takes place. Secondly, targeting the LRP1 receptor could enrich the presence of the inhibitor in the proximity of BACE1, facilitating their interaction. Finally, the spatial confinement of the inhibitor in the endosomes could potentially spare the off-target effects associated with the indiscriminate inhibition of other BACE1 substrates’ processing in other cellular compartments^21^,^22^. This could be tested by monitoring the inhibitor’s impact on the cleavage of another substrate of BACE1, such as Neuregulin-1^23^,^24^. Furthermore, the expression pattern of the LRP1 receptor in neurons and brain endothelial cells could potentially enable the delivery of the drug across the BBB.

Numerous ligands have been described for LRP1, reflecting its many physiological functions. The diverse list of ligands ranges from intracellular proteins like the receptor-associated protein (RAP), to matrix proteins, growth factors and others like alpha-macroglobulin, aprotinin and APP itself^19^. Some of these ligands share a common sequence called the Kunitz proteinase inhibitor (KPI) domain, which quite expectedly was found to be important for LRP1-mediated endocytosis^25^. Based on the sequence of the KPI domain and of other LRP1 ligands, a transport peptide called Angiopep-2 (ANG) was developed and proposed for the delivery of molecules to brain tissue. Its enhanced transcytosis in an *in vitro* model of the BBB was attributed to its interaction with the LRP1 receptor^26^,^27^. Shortly after its development, the first example of ANG-mediated delivery of paclitaxel was reported and clinical trials were carried out^28^.

In this work, we have coupled the transport peptide ANG to a peptidic β-secretase inhibitor in order to construct a peptidic bioconjugate with the potential to engage in endocytosis *via* interaction with the LRP1 receptor, concentrate in the endosomes of neurons and inhibit the activity of BACE1 selectively in this intracellular compartment. To this end, an octapeptidic β-secretase inhibitor based on the transition-state analogue statine and with sequence EVN-statine-VAEF was selected^29^. For the sake of target specificity, the choice of inhibitor was mainly based on its hydrophilicity and cell-impermeability rather than its potency (although its IC50 is within a range comparable to other inhibitors undergoing clinical trials)^3^,^30^, so that the conjugate would be delivered intracellularly and become active only upon coupling to the ANG moiety^29^.

## Results

### Cellular internalization of ANG-SI and accumulation with enzyme in endosomal compartment

**Figure 1:**
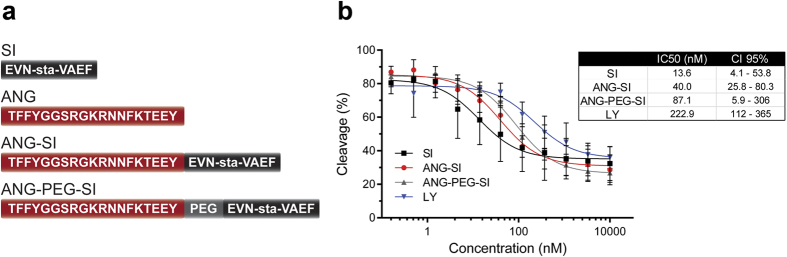
Peptidic bioconjugates and their activity on purified BACE1 enzyme. (**a**) Scheme of the main bioconjugates used in this study with their corresponding sequences. (**b**) *In vitro* inhibitory activity of the peptidic inhibitor SI^29^, the conjugates ANG-SI and ANG-PEG-SI, and the non-peptidic inhibitor LY (LY2811376, Eli Lilly) on a cell-free assay of purified BACE1 enzyme (mean ± SD, n = 3 except for ANG-PEG-SI where n = 2). The IC50 values calculated from the experimental data with the corresponding confidence intervals (CI 95%) represent the concentrations in nM at which 50% of relative cleavage was achieved with each bioconjugate.

We selected a statine-based, transition-state, octapeptide β-secretase inhibitor (SI) with binding affinity K_i_ 33 nM in a cell-free system^29^,^31^. The hydrophilic nature of the SI prevents it from overcoming membranes and entering cells, making it inactive on cellular systems. We coupled the SI to the transport peptide ANG rendering the conjugate ANG-SI (Fig. 1a). In line with what was previously described, modifying the N-terminal of the SI by coupling the ANG peptide did not have a significant impact on its potency, as shown by the concentration-dependent inhibition of the enzyme in a cell-free assay (Fig. 1b). In addition, since the distance between the targeting and inhibitor moieties has been shown to affect the efficacy of secretase inhibitory complexes^32^, we introduced between ANG and SI an inert linker of poly(ethylene glycol) (PEG) of a specific length that was previously reported to be functional in a similar system^14^. Although the potency of the resulting ANG-PEG-SI conjugate was lower than that of the other conjugates, it was nevertheless included in subsequent experiments based on the potential enhancement that the flexible linker could bring when tested in cellular systems. The potency of the non-peptidic inhibitor LY (LY2811376, Eli Lilly) against the purified enzyme was also measured, as this small molecule was later on used as a positive control for BACE1 inhibition in cellular systems given its cell permeability.

**Figure 2:**
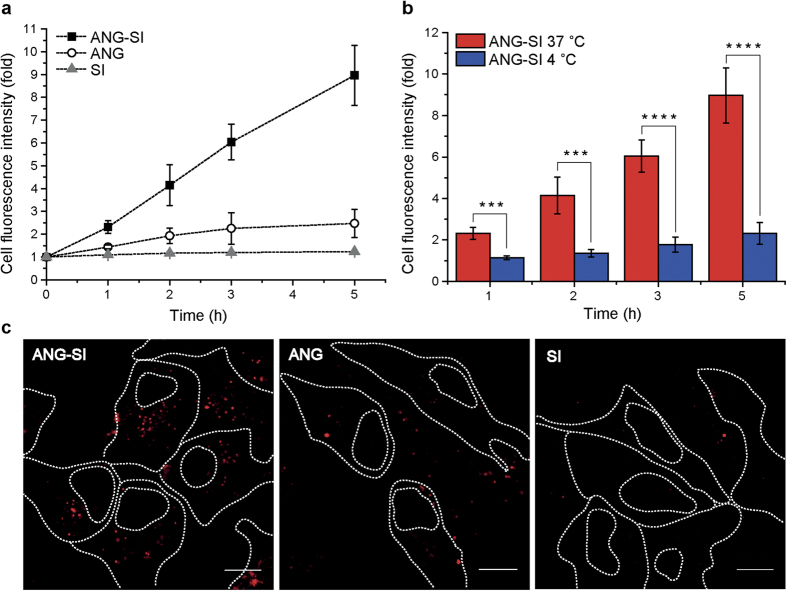
Cellular internalization of peptidic bioconjugates in SH-SY5Y neurons. Mean cell fluorescence intensity was measured by flow cytometry after continuous incubation with the fluorescent TAMRA-labeled bioconjugates in FBS-supplemented medium for increasing time periods. Values were normalized against the fluorescence background of untreated cells and expressed as fold increase. (**a**) Internalization kinetics of ANG-SI, ANG or SI at 1 μM (mean ± SD, n = 3). (**b**) Energy-dependence of the internalization of ANG-SI at 37 °C and 4 °C (mean ± SD, n = 3). ***P ≤ 0.001 and ****P ≤ 0.0001, unpaired Student’s *t* test. (**c**) Confocal images of cells incubated with ANG-SI, ANG or SI (red) for 1 h. Cells’ perimeter and nuclei have been delineated for reference. Scale bar 10 μm.

The human neuroblastoma cell line SH-SY5Y was incubated in presence of fluorescent versions of the SI inhibitor, the ANG-SI conjugate and the ANG peptide labeled with the dye TAMRA (5- carboxytetramethylrhodamine) and their internalization was monitored by flow cytometry and confocal microscopy. The gating used to exclude cell debris, aggregates and non-viable cells in the analysis of flow cytometry data can be found in Supplementary Fig. S1. As expected, the hydrophilic SI alone did not enter cells during the course of the 5 h incubation period, while ANG and ANG-SI were taken up by cells as indicated by the increasing cell fluorescence intensity (Fig. 2a). The ANG-SI conjugate was internalized with markedly higher efficiency than the ANG peptide itself, an observation that has been previously reported in other studies involving ANG conjugates^33^,^34^. We corroborated that the difference in uptake efficiency was not due to differences in the constructs labelling efficiency with TAMRA dye as confirmed by fluorescence intensity measurements of the stocks (Supplementary Fig. S2) and purity analysis by liquid chromatography–mass spectrometry (LC-MS) (Supplementary Fig. S3). The internalization of the ANG-SI conjugate was significantly decreased when incubation was performed at 4 °C instead of at 37 °C, which is indicative of an energy-dependent uptake mechanism (Fig. 2b). Confocal imaging confirmed the intracellular localization of the ANG-SI conjugate, revealing a punctate pattern typical of vesicular accumulation. In contrast, the SI alone was not detectable on top nor inside the cells, confirming the molecule’s inability to gain cellular access (Fig. 2c). Interestingly, the difference in uptake efficiency between ANG and the ANG-SI conjugate was also evident in the microscopy images, which showed that intracellular accumulation was much higher with the latter. It is possible that differences in the organization of the two sequences in solution would influence their capacity to establish hydrophobic and electrostatic interactions with the cell membrane, ultimately affecting their uptake efficiency.

**Figure 3:**
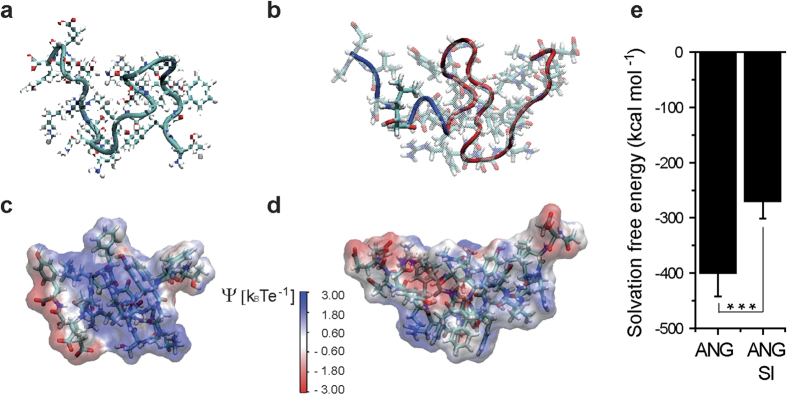
ANG and ANG-SI structures from molecular dynamics simulations. Metadynamics simulations were employed to obtain folded geometry in solution, while classical molecular dynamics simulations were carried out for structural analysis. Representative folded structures in solution of (**a**) ANG and (**b**) ANG-SI are shown, with the SI colored in blue and the ANG in red. Note that the statin amino acid (opaque residue) is well exposed on the peptide surface. Electrostatic potentials on the surfaces of (**c**) ANG and (**d**) ANG-SI are depicted with blue surfaces representing positively charged areas and red surfaces negatively charged areas. (**e**) Mean solvation free energy for ANG and ANG-SI. ***P ≤ 0.001, unpaired Student’s *t* test.

To explore this hypothesis, molecular dynamics simulations were employed and the potential effect of protein conformation on the internalization efficiency of ANG and ANG-SI was evaluated. Since crystallographic structures representative of the folded conformation of ANG and ANG-SI molecules in water were not available in literature, metadynamics simulations were first performed in order to sample peptide structures (Supplementary Fig. S4) and obtain representative folded geometries in solution (Fig. 3a,b)^35^. Subsequently, 50 ns molecular dynamics simulations were carried out for both folded ANG and ANG-SI, while the last 10 ns were used for analysis of molecular trajectories (Supplementary Fig. S5). All simulations were performed with explicit water molecules and explicit Na^+^ and Cl^−^ ions, whose concentrations mimicked PBS environment. In line with the potent inhibitory activity observed for the ANG-SI conjugate in the cell-free assay of purified BACE1 enzyme, the statin residue in the inhibitory sequence was found to be well exposed on the peptide surface.

The electrostatic potential Ψ on ANG and ANG-SI surfaces was calculated and expressed in dimensionless k_B_Te^−1^ unit where k_B_ is the Boltzmann constant, T is the absolute temperature and e is the electron charge (Fig. 3c,d)^36^. The net result was the presence of large positively charged areas on ANG surface, while for ANG-SI charged groups were less exposed to the solvent. This is not surprising since the overall charge of ANG at pH 7.4 is equal to +2, whereas that of the ANG-SI conjugate is equal to 0. Moreover, the specific charge distribution observed in ANG makes the peptide more hydrophilic in comparison to the ANG-SI conjugate, as supported by the solvation free energy which is more favorable for the former (Fig. 3e)^37^. Thus, the obtained results suggest that non-specific hydrophobic interactions can play a relevant role in ANG-SI internalization.

**Figure 4:**
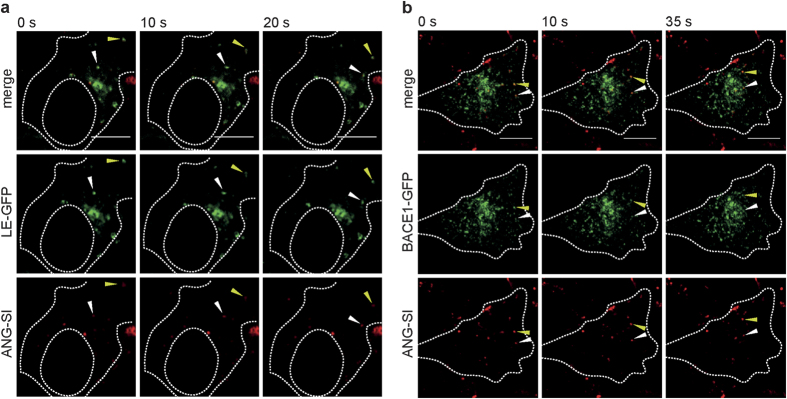
Intracellular fate of the ANG-SI bioconjugate in SH-SY5Y cells. Confocal images of SH-SY5Y cells expressing either (**a**) a GFP-tagged marker for late endosomes (green), or (**b**) a GFP-tagged BACE1 (green), exposed to ANG-SI (red) for 1 h, washed with fresh FBS-supplemented medium and imaged every 5 sec over 2 min. Panels show images of the same cell at different time intervals, at which GFP-positive vesicles can be observed moving together with a load of ANG-SI (the trajectory of two different vesicles is indicated with white and yellow arrows). The perimeter and nucleus of the cells have been delineated for reference. For the complete time-lapse videos see Supplementary videos SV1-6. Scale bar 10 μm.

We then analyzed the intracellular fate of the ANG-SI conjugate using high-speed live-cell confocal imaging. Since the punctate pattern of ANG-SI intracellular accumulation suggested its presence in endocytic vesicles, SH-SY5Y cells were transfected to express GFP-tagged Rab7a, a marker of late endosomal vesicles (LE-GFP). We observed that upon incubation with fluorescently labeled ANG-SI, some of the LE-GFP-positive vesicles of the transfected cells were also positive for ANG-SI (Fig. 4a and Supplementary videos SV1–3). Although there was only a partial overlap between the labeled conjugate and the vesicles, some of the double-positive vesicles were found to move actively inside the cell during the time-resolved acquisition, suggesting the presence of the conjugate in this subset of endocytic vesicles. Following the same approach, we then studied SH-SY5Y cells transfected to express fluorescent BACE1 enzyme (BACE1-GFP) and found that the ANG-SI conjugate associated with some of the labeled enzyme as indicated by their co-movement inside of the cells (Fig. 4b and Supplementary videos SV4–6). Representative snapshots of the sequences of images acquired every 5 s over 2 min are shown and the arrows indicate the active movement of intracellular vesicles that are simultaneously positive for the ANG-SI conjugate and the endosomal marker or the target enzyme BACE1. The full videos from which the snapshots were extracted as well as additional videos can be found in the Supplementary material (see Supplementary videos SV1–6). Hence, ANG-SI is efficiently internalized by neuroblastoma cells, where it accumulates inside endosomal vesicles together with the target enzyme BACE1.

### Inhibitory activity of ANG-SI in neurons

**Figure 5:**
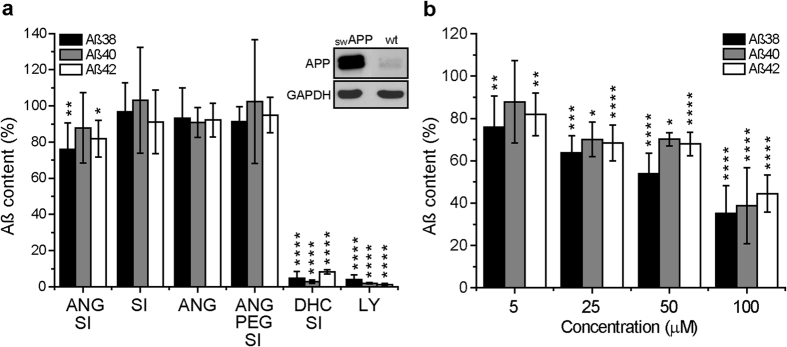
BACE1-inhibitory activity of the bioconjugates. (**a**) Inhibitory activity of ANG-SI, SI, ANG, ANG-PEG-SI, DHC-SI and LY2811376 (LY) at 5 μM after 6 h of continuous incubation in FBS-supplemented medium on Swedish-mutant APP-overexpressing (swAPP) SH-SY5Y cells. The content of Aβ peptides was measured in the cells’ supernatant upon treatment and normalized setting the values of control DMSO-treated cells as 100% (Mean ± SD, n = 3–6). Inset shows Western blots indicating the level of expression of APP in swAPP and wild type (wt) SH-SY5Y cells. GAPDH was used as loading control. (**b**) Inhibitory activity of the ANG-SI bioconjugate at a range of concentrations (5–100 μM) after 6 h incubation with swAPP cells (Mean ± SD, n = 3–6). *P ≤ 0.05, **P ≤ 0.01, ***P ≤ 0.001, ****P ≤ 0.0001, one-way ANOVA followed by Dunnet test.

Based on earlier work in which the coupling of dihydrocholesterol (DHC) to the same SI that we used here blocked BACE1 activity as a consequence of successful delivery of the complex to the membrane^14^, we studied whether the endosomal localization of the ANG-SI conjugate in the close vicinity of BACE1 would also enable the inhibition of the enzyme. The inhibitory activity of the different ANG conjugates was evaluated by measuring the products of APP cleavage (Aβ peptides 38, 40 and 42) by enzyme-linked immunosorbent assay (ELISA) in the cell cultures’ supernatant after incubating them for 6 h with the inhibitors at 5 μM. The amounts detected varied among the different Aβ peptides (Aβ42 ~70,000 RU, Aβ40 ~400,000 RU and Aβ38 ~4,000 RU) and were therefore normalized for the basal Aβ levels measured in the supernatant of DMSO-treated cells, which were set as 100%. Since the levels of Aβ peptides released by wild-type SH-SY5Y cells were close to the limit of detection of the ELISA kit we employed, we used instead SH-SY5Y cells overexpressing Swedish-mutant APP (swAPP)^38^ to assess the conjugates’ activity. As positive controls we used the cell-permeable non-peptidic inhibitor LY2811376 and the previously described sterol-linked conjugate DHC-SI. Whereas the positive controls efficiently decreased the levels of all three Aβ peptides in the supernatant of swAPP SH-SY5Y cells (>90% decrease), the effect of the ANG conjugates was negligible at the dose applied (Fig. 5a). The ANG-PEG-SI conjugate did not show cellular activity either, although it was expected to benefit from a higher degree of freedom to interact with BACE1 inside the endosomal vesicles. Comparable results were also obtained with two other SH-SY5Y cell lines stably overexpressing the isoforms APP_695_ and APP_751_ (Supplementary Fig. S6)^39^. Moreover, similar observations were made with conjugates prepared with other well-known transport peptides such as the trans-activating transcriptional activator (TAT) from the human immunodeficiency virus 1 (HIV-1) and transportan (TRP) from the neuropeptides galanine/mastoparan (Supplementary Fig. S7), both of which have been shown to be largely internalized through receptor-independent pathways^40^,^41^,^42^. The TRP-SI conjugate decreased the levels of Aβ peptides marginally, but the effect was related to its cytotoxic effect (Supplementary Fig. S8). Interestingly, the ANG-SI conjugate induced a modest yet statistically significant ~25% decrease in the enzymatic activity of BACE1 at concentrations five times higher, and decreased the activity by ~60% at concentrations twenty times higher (Fig. 5b). Although rather mild, the inhibitory effect was only observed with the ANG-SI conjugate and not the ANG or SI peptides alone (Supplementary Fig. S9), indicating the benefit of coupling the ANG transport peptide as compared to using the unmodified inhibitor.

Therefore, in spite of the successful peptide-aided delivery of the inhibitor and its association with the target enzyme observed during active vesicle trafficking, high doses of the ANG-SI conjugate were necessary to induce a detectable decrease in the activity of BACE1. Since the conjugate’s potency against the purified enzyme was comparable to that of DHC-SI and LY2811376, the low efficiency of ANG as a transport peptide is possibly the limiting factor in the effect achieved at a cellular level.

### Involvement of the LRP1 receptor in the uptake of ANG and ANG-SI

**Figure 6:**
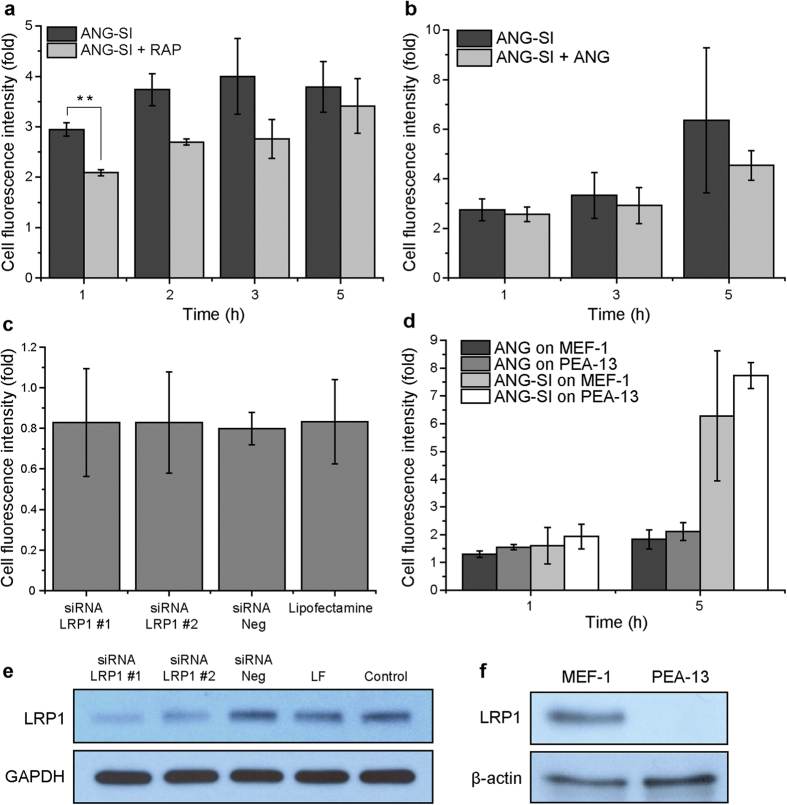
Involvement of the LRP1 receptor in the cellular internalization of the ANG-SI bioconjugate. (**a**) Mean cell fluorescence intensity after continuous incubation with ANG-SI at 1 μM in absence or presence of the LRP1-blocking ligand RAP at 1 μM. **P ≤ 0.01, unpaired Student’s *t* test. (**b**) Mean cell fluorescence intensity after continuous incubation with ANG-SI at 1 μM in absence or presence of a 10-fold excess of non-fluorescent ANG peptide. (**c**) Mean cell fluorescence intensity after 4 h of incubation with ANG-SI at 1 μM. Cells were previously transfected with siRNA for LRP1 (siRNA LRP1 #1 or #2), scrambled siRNA (siRNA Neg) or lipofectamine alone. The corresponding Western blot showing the knockdown of LRP1 by siRNA is shown below; GAPDH was used as loading control (**e**). (**d**) Mean cell fluorescence intensity of MEF-1 cells (LRP1^+/+^) or PEA-13 cells (LRP1^−/−^) incubated with ANG-SI or SI at 1 μM. The Western blot for LRP1 corresponding to the two cell lines is shown below; β-actin was used as a loading control (**f**). Numerical data are represented as mean ± SD (n = 3).

Considering the tripartite interaction between LRP1, BACE1 and APP^15^,^20^ and the described role for LRP1 in the uptake of ANG^27^, we analyzed in more detail the internalization process of the ANG-SI conjugate to gain mechanistic insights regarding its low potency. In general, the involvement of a receptor in a ligand’s endocytosis process can be tested using a competing molecule that binds to it and thus interferes with the ligand’s uptake. We conducted competition experiments in which we pre-incubated SH-SY5Y cells with RAP (a known high-affinity ligand of the LRP1 receptor) or the unconjugated ANG peptide to occupy the LRP1 receptor’s binding sites and compete against the uptake of the ANG-SI conjugate^18^. Whereas the presence of a 10-fold molar excess of ANG did not affect the uptake level of ANG-SI, the presence of equimolar amounts of RAP decreased it, although only the earliest time point tested was statistically significant (Fig. 6a,b). As negative control ligands we used two LRP1-independent molecules, transferrin and dextran, which were internalized with the same efficiency in the presence or absence of RAP and ANG, as expected (Supplementary Fig. S10). While our results suggested at best a marginal involvement of the receptor, previous work on ANG and LRP1 reported much stronger effects during competition experiments^27^. In contrast to our protocol, such studies employed an excess of ANG peptide to compete against the uptake of RAP. Moreover, RAP was used at 250 nM and a 100-fold excess of ANG was added to compete against it, meaning that the peptide was used at 25 μM, which is 25 times higher concentration than what we used. Further differences could arise from the use of radiolabeling instead of fluorescence as was our case, and from the cells used, which were mouse embryonic fibroblasts instead of human neuroblastoma cells.

Since our competition experiments were inconclusive, we further studied the uptake of ANG-SI while varying the expression levels of LRP1. First, we knocked down its expression using LRP1-targeting siRNA in SH-SY5Y cells. Strikingly, the marked decrease of LRP1 observed by Western blot led to essentially no differences in the uptake efficiency of ANG-SI (Fig. 6c,e). We then measured the uptake of the conjugate in complete absence of the receptor, using mouse embryonic fibroblasts genetically deficient for the LRP1 gene (PEA-13 cells) (Fig. 6d,f)^43^. Interestingly, the absence of the receptor did not have any impact on the uptake efficiency and kinetics of ANG-SI nor ANG, since the wild-type counterpart of these murine cells in which LRP1 is normally expressed (MEF-1 cells) showed similar results. Thus, while for other cell lines the interaction between ANG and LRP1 has been found to be at least partly responsible for the peptide’s internalization^27^, in the cell lines used here it did not appear to be essential.

### Uptake mechanism of ANG in SH-SY5Y cells

Given that the interaction with the LRP1 receptor did not appear to be the main mechanism for ANG’s internalization in SH-SY5Y cells, we systematically inhibited different endocytic pathways using classical chemical inhibitors to obtain further information on the underlying mechanism. Control experiments with relevant endocytosis markers were carried out and cell viability was closely monitored (Supplementary Fig. S11). We used the small cell-permeable molecule Dynasore to inhibit the GTPase activity of dynamin, which is necessary for the formation of clathrin-coated vesicles^44^. Fluorescently labeled human transferrin was used to verify the inhibition of clathrin-mediated endocytosis by Dynasore. The amiloride derivative 5-(N-Ethyl-N-isopropyl)amiloride (EIPA), which inhibits Na^+^/H^+^ exchange, was used to block macropinocytosis^45^. We monitored this effect with the fluorescent fluid phase marker dextran (70 kDa).

**Figure 7:**
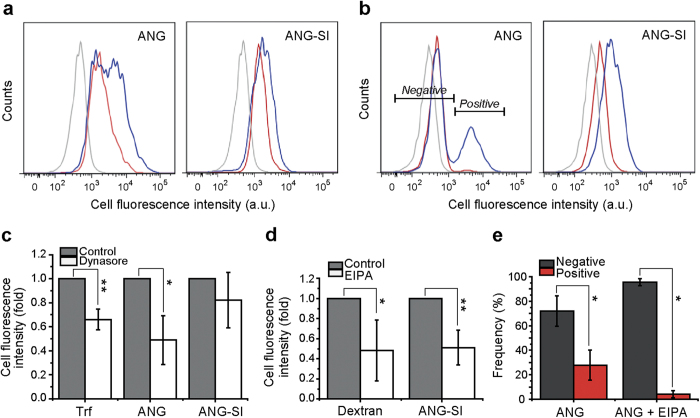
Pharmacological inhibition of the uptake of ANG and ANG-SI. Cell fluorescence distributions of cells treated with **(a)** 100 μM Dynasore or (**b**) 60 μM EIPA. After 30 min pre-treatment with inhibitor or medium alone, the cells were incubated for 1 h with 3 μM ANG or ANG-SI in presence (red) or absence (blue) of the inhibitor used in the pre-treatment. Background cell fluorescence of only inhibitor-treated cells is shown (grey). Distributions shown are representative examples of three independent experiments. (**c,d**) Mean cell fluorescence intensity of the distributions shown in (**a**) or (**b**), respectively. The markers transferrin (Trf) and Dextran are shown for reference. (**e**) Frequency of cells in each of the two subpopulations (*Negative* or *Positive*) present upon treatment with ANG, as defined by the gates shown in the fluorescence distribution in (**b**). Numerical data are represented as mean ± SD (n = 3). *P ≤ 0.05 and **P ≤ 0.01, one-way ANOVA followed by Dunnet test.

SH-SY5Y wild-type cells were pre-treated for 30 min with the endocytosis inhibitors, followed by incubation with the corresponding fluorescent marker (Supplementary Fig. S12) or peptide in presence or absence of the same inhibitor. Treatment with 100 μM Dynasore was done in serum-free medium to prevent activity loss as a consequence of interaction with serum proteins. In the presence of Dynasore, the uptake of the clathrin-mediated endocytosis marker transferrin was decreased by ~35%. Under the same conditions, the uptake of ANG peptide was decreased by 50% while that of the ANG-SI conjugate decreased only by 20% (Fig. 7a,c). The fact that the internalization of the unconjugated peptide was more affected than that of the ANG-SI conjugate would indicate that the former is dependent on dynamin to a larger extent, implying that they could be exploiting different uptake mechanisms. Treatment with 60 μM EIPA lowered the uptake of the macropinocytosis marker dextran and of ANG-SI by 50% (Fig. 7b,d). For the unconjugated ANG peptide however, two clear populations could be observed in the cell fluorescence distribution (Fig. 7b, left). Interestingly, EIPA did not affect the mean fluorescence intensity but rather the frequency of cells in each subpopulation (Fig. 7e). We defined “Negative” and “Positive” subpopulations depending on their fluorescence intensity and found that the frequency of the latter subpopulation decreased from ~30% to ~4%, with a corresponding increase in the former subpopulation. This would suggest firstly that the uptake efficiency of ANG is greater within one subset of cells and secondly, that the internalization of peptide in such subpopulation would be macropinocytosis-mediated while EIPA-insensitive in the other.

Hence, pharmacological inhibition of the different endocytosis pathways revealed that the unconjugated ANG peptide is probably internalized through a combination of endocytic pathways which differ from those exploited by the ANG-SI conjugate. In other words, the transport peptide ANG could engage in different endocytosis mechanisms depending on the moiety to which it is conjugated. Far from being an isolated case, similar observations have been previously described for other well-known transport peptides such as TAT. Unconjugated TAT was shown to gain intracellular access *via* clathrin-dependent endocytic mechanisms^46^. However, upon its conjugation with large fusion proteins, it could be internalized instead by a lipid raft-dependent^47^ kind of fluid-phase macropinocytosis^48^,^49^.

## Discussion

The development of efficient secretase inhibitors with the capacity to decrease the neurotoxic aggregates of Aβ peptide characteristic of Alzheimer’s disease has been hindered by their lack of specificity, which often leads to inadmissible off-target consequences. Encouraging reports of efficient inhibition of BACE1 upon membrane targeting of a sterol-modified inhibitor, motivated us to design conjugates with the potential to engage in receptor-mediated endocytosis and be active in a spatially confined manner, sparing unintended off-target inhibition of other substrates’ processing by BACE1. To this end, the transport peptide ANG was coupled to a hydrophilic secretase inhibitor and its interaction with neuroblastoma cells and with the activity of BACE1 was investigated.

Despite being efficiently internalized by neurons and concentrating in the endosomal compartment where the target enzyme is most active due to the low pH, high doses of the ANG-SI conjugate were required to inhibit BACE1’s activity to a significant extent. Given that its *in vitro* potency against purified BACE1 was comparable to the one of the other effective inhibitors tested, its lack of efficacy on cellular systems is probably due to the fact that the dose of inhibitor achieved in the endosomes is not sufficient to trigger a significant response on cells. This could in turn be related to the finding that LRP1 receptor-mediated endocytosis did not contribute to the conjugate’s uptake to a detectable extent in the cell lines we used. However, we cannot exclude the potential influence that coupling the SI to the ANG peptide may exert on its targeting efficacy. Indeed, pharmacological inhibition experiments indicated that the mechanism through which ANG gains cellular access might change upon conjugation to the SI moiety. This finding underlines the importance of corroborating the delivery function of transport peptides as well as their uptake route upon conjugation to drugs.

Previously published work on the sterol-modified inhibitor conjugate indicated that targeting these drugs to the membrane to render them endocytosis-competent would be a pre-requisite for their cellular activity. In such work, the inhibitor conjugate benefited from insertion into the membrane plane and enrichment in sterol-rich domains. Our data suggest that using a transport peptide such as ANG to promote the inhibitor’s endocytosis and shuttle it to the correct intracellular organelle might not be efficient enough to modulate the enzymatic activity of BACE1. It is likely that targeting a specific microdomain in the plasma membrane and/or a receptor like LRP1 to which the target enzyme and/or substrate is associated, could improve the inhibitor’s efficacy. However, according to our results, the interaction with the LRP1 receptor is not essential for the ANG peptide’s intracellular access in the cell lines tested.

## Materials and Methods

### Peptides

All peptides were custom-made by and purchased from Peptide 2.0 (Chantilly, VA), except ANG-PEG-SI and DHC-SI which were obtained from Cambridge Research Biochemicals (Cleveland, UK) and American Peptide Company (Sunnyvale, CA), respectively. Stock solutions were prepared at 1 mM in DMSO and stored at −80 °C. The peptides’ sequences are as follows: ANG (TFFYGGSRGKRNNFKTEEY), SI (EVN-statine-VAEF), ANG-SI (EVN-statine-VAEFTFFYGGSRGKRNNFKTEEY), ANG-PEG-SI (EVN-statine-VAEF-PEG-TFFYGGSRGKRNNFKTEEY) where PEG is 4Gl-3Gl-4Gl-4Gl-4Gl, DHC-SI (EVN-statine-VAEF-PEG-D(DHC)-NH_2_) where DHC is dihydrocholesterol, TAT-SI (EVN-statine-VAEFRKKRRQRRR), TRP-SI (EVN-statine-VAEFGWTLNSAGYLLGKINLKALAALAKKIL). Fluorescent versions of the peptides labeled with TAMRA dye (5-carboxytetramethylrhodamine) were also obtained from Peptide 2.0 and their purity and molecular weight were confirmed by liquid chromatography–mass spectrometry (LC-MS) using a XBridge C18 5 μm column with a gradient 25–45% B in 15 min (where buffer A was 0.05% trifluoroacetic acid in mQ H_2_O and buffer B was 0.05% trifluoroacetic acid in 90% acetonytrile).

### Cell lines

SH-SY5Y human neuroblastoma cells (CRL-2266), PEA-13 (CRL-2216) and MEF-1 (CRL-2214) mouse embryonic fibroblasts were purchased from ATCC (Manassas, VA). The cell lines were grown in DMEM supplemented with 10% FBS and 1% penicillin-streptomycin at 37 °C and 5% CO_2_. All cell culture reagents were purchased from Life Technologies-ThermoFisher Scientific (Waltham, MA). SH-SY5Y cells over-expressing the Swedish mutant APP were obtained from Prof. Charles Duyckaerts at the ICM Brain & Spine Institute, Paris. SH-SY5Y cells stably expressing the APP_695_ and APP_751_ isoforms were kindly provided by Prof. Nigel Hooper at University of Manchester, UK.

### Flow cytometry

Unless otherwise stated, for flow cytometry-based experiments cells were seeded in 24-well plates (80,000 cells/well) one day before carrying out the experiments to allow cells’ adherence. A Becton-Dickinson FACS Canto cytometer (Franklin Lakes, NJ) was used, followed by analysis with the FlowJo software. For data analysis, the gating shown in Supplementary Fig. S1 was applied to separate cell debris and aggregates. To corroborate that the gating used excluded non-viable cells, the LIVE/DEAD Viability/Cytotoxicity kit from ThermoFisher Scientific (Waltham, MA) was used following manufacturer’s guidelines.

To study the uptake of the ANG conjugates, cells were incubated with the different fluorescently labeled peptides at 1 μM in FBS-supplemented medium for increasing periods of time, after which they were washed according to previously established protocols^50^ and harvested with trypsin for their analysis by flow cytometry. The mean fluorescence values obtained from the analysis were normalized against the background fluorescence of untreated cells and were presented as fold-increase of this intensity.

To study uptake in energy-depletion conditions, the cells were pre-incubated for 1 h at 4 °C, followed by incubation with the fluorescent peptides in FBS-supplemented medium for increasing periods of time at 4 °C as well. The cells were then washed and harvested for their analysis by flow cytometry.

For competition experiments, the uptake of fluorescent ANG-SI peptide was measured after different incubation times in presence or absence of 1 μM RAP (Oxford Biomedical Research; Rochester Hills, MI) or 10 μM unlabeled ANG. The rationale behind this experiment is that if the presence of competitive LRP1-binding ligands such as RAP or ANG hampers the uptake of ANG-SI, then the LRP1 receptor is involved in the conjugates’ internalization. To evaluate potential unspecific effects of RAP on non-LRP1-mediated endocytic processes, we also measured in the presence of RAP the uptake of molecules whose internalization is known to be independent of LRP1. For this purpose we used 0.125 μM fluorescently labelled human transferrin (which exploits the transferrin receptor) and 0.8 mg/mL FITC-labelled 70 kDa Dextran (which enters cells via macropinocytosis), both purchased from Sigma-Aldrich (St Louis, MO).

For the pharmacological inhibition of endocytosis, we used Dynasore to inhibit clathrin-mediated endocytosis and 5-(N-Ethyl-N-isopropyl)amiloride (EIPA) to block macropinocytosis. Both reagents were purchased from Sigma-Aldrich (St Louis, MO). To verify their inhibitory effects, we used fluorescently labeled human transferrin and 70 kDa dextran, respectively. Cells were seeded in 24-well plates at a lower cell density (40,000 cells/well) and on the next day pre-treated for 30 min with 100 μM Dynasore or 60 μM EIPA, followed by 1 h incubation with 5 μg/mL fluorescently labeled human transferrin, 0.75 mg/mL 70 kDa Dextran, 3 μM ANG or 3 μM ANG-SI in presence or absence of the same inhibitor used in the pre-treatment. Dynasore-treated cells were washed thrice with acidic wash buffer (0.2 M acetic acid and 0.2 M NaCl) and once with PBS prior to being harvested with trypsin for their analysis by flow cytometry. EIPA-treated cells were washed once with PBS, twice with acetate wash buffer (0.1 M sodium acetate and 0.05 M NaCl, pH 5.5) for 5 min each, and finally once more with PBS prior to being harvested with trypsin for their analysis by flow cytometry.

### Downregulation of LRP1

Cells were seeded in 24-well plates (50,000 cells/well) and transfected on the next day for 24 h with 1.5 mg/mL Lipofectamine2000 and 0.5 mg/mL siRNA, after which the cells were washed and grown further for 48 h to allow for gene downregulation. Upon verification of the knockdown effect by Western blotting (see below), the uptake of fluorescent ANG-SI was measured as described above. For LRP1 downregulation the siRNA #8279 and #8280 were used. As a scrambled negative control, a siRNA against a mouse sequence was used. All transfection reagents were purchased from Life Technologies-ThermoFisher Scientific (Waltham, MA).

### Microscopy

To visualize the intracellular accumulation and localization of the fluorescent ANG conjugates, cells were seeded in 4-well chambered slides (LabTek, 100,000 cells/well) and transfected on the following day for 6 h with either Late Endosome CellLight from ThermoFisher Scientific which labels the Rab7a protein (Waltham, MA) or BACE1-GFP from OriGene (Rockville, MD), after which the cells were washed and grown for another day to enable protein expression. Cells were then incubated with 3 μM fluorescent ANG-SI, ANG or SI in FBS-supplemented medium for 1 h, after which they were washed with PBS and imaged live at 37 °C and 5% CO_2_ using a Zeiss Spinning disk confocal microscope equipped with 488 and 633 nm lasers. Images were acquired every 5 sec for 2 min. The time-lapse videos acquired for each staining can be found in the Supplementary material, while Fig. 4 shows snapshots of these videos. Image analysis was carried out using ImageJ software.

### Western Blots

The expression level of LRP1 and APP were measured by Western blot. Briefly, protein extracts were obtained from whole cell lysates and quantified with the Pierce BCA assay from ThermoFisher Scientific (Waltham, MA). For SDS-PAGE 10% gels were used, 10 μg of protein sample was loaded and electrophoresis was run at 150 mV for 70 min. Protein transfer to a previously methanol-activated PVDF membrane was carried out at 400 mA for 1 h at 4 °C. Membranes were blocked in blocking buffer (5% milk in TBS-Tween20) for 1 h at room temperature with shaking. Overnight incubation with primary antibodies in blocking buffer was done at 4 °C with shaking. After four 5-min TBS-Tween20 washes, membranes were incubated with secondary antibodies in blocking buffer for 4 h at room temperature with shaking. After another round of four 5-min TBS-Tween20 washes, membranes were developed with X-ray films and ECL reagent from Santa Cruz Biotechnology (Heidelberg, Germany). The antibodies and dilutions used were as follows: LRP1 1:20,000 (Abcam #ab92544) (Cambridge, UK), APP 1:1,000 (Abcam #ab15272), GAPDH 1:2,000 (Santa Cruz #sc-47724), Beta-actin 1:5,000 (Abcam #ab8227), HRP-secondary goat anti-rabbit 1:5,000 (Abcam #ab6721), HRP-secondary goat anti-mouse 1:5,000 (Dako-Agilent Technologies #P0447) (Santa Clara, CA).

### Activity test against purified BACE1 enzyme

A range of concentrations of the different bioconjugates were tested in a cell-free assay of purified BACE1 enzyme using the SensoLyte 520 β–secretase assay kit from AnaSpec (Fremont, CA) following manufacturer’s guidelines.

### Activity test on human neurons

SH-SY5Y cells overexpressing either Swedish-mutant APP or the isoforms APP_695_ or APP_751_ were seeded in 24-well plates (100,000 cells/well) and after one day they were washed twice with FBS-supplemented medium and incubated for 6 h with the inhibitor LY2811376 from Eli Lilly (Indianapolis, IN) or the different bioconjugates (SI, ANG-SI, ANG-PEG-SI, DHC-SI, TAT-SI, TRP-SI) at 5 μM. Supernatant from the tissue culture plates was then collected in low protein-binding tubes and analyzed by enzyme-linked immunosorbent assay with the Multi-Spot Assay System from Meso Scale Discovery (Rockville, MD) detecting human Aβ38, Aβ40 and Aβ42 peptides (#K15200E,) following manual’s instructions. The values obtained for the different conjugates varied in intensity (Aβ42 ~70,000 RU, Aβ40 ~400,000 RU and Aβ38 ~4,000 RU) and were normalized for the basal Aβ level detected in the supernatant of DMSO-treated cells, which was set as 100%.

### Statistical analysis

Results were compared using unpaired Student’s *t* test, except in the cases where three or more independent groups were compared (Figs 5, 6c, S5, S6 and S8), for which one-way ANOVA followed by a post-hoc test (Dunnett) was used to identify the groups having statistically significant difference against the control group. In these cases ANOVA was preferred over multiple t-tests in order to reduce the Type I error probability (α-inflation)^51^. *P* values lower than 0.05 were considered to be significant. Statistical analyses were carried out using GraphPad Prism software.

### Computational methods

Representative geometries of the folded ANG and ANG-SI peptides in solution were generated by metadynamics simulations, given that their crystallographic structures were not available in literature^35^. Broadly speaking, metadynamics simulations allow obtaining the free energy of the system of interest as a function of few relevant degrees of freedom (collective variables). In this case, the radius of gyration of α-carbon atoms in the peptide backbone and intrapeptide hydrogen bonds were chosen as collective variables since they can provide a good sampling of protein structures in solution^52^. Molecular dynamics simulations were then performed in order to further analyze the folded structures of ANG and ANG-SI. The peptides were solvated with explicit water molecules, and explicit ions of Na^+^ and Cl^−^ were added to achieve electroneutrality and mimic the PBS environment. Subsequently, 50 ns molecular dynamics simulations were carried out at constant pressure and temperature (1 atm and 310 K, respectively). The last 10 ns were employed for the analysis of molecular trajectories. Electrostatic potentials were computed by means of Adaptive Poisson Boltzmann Solver (APBS)^36^ and were expressed in dimensionless kBTe-1 units where kB is Boltzmann constant, T is the absolute temperature and e is electron charge. Mean solvation free energy was calculated by means of MMPBSA as implemented in AmberTools package^37^. A detailed description of the simulations protocols can be found in the Supporting Information.

## Additional Information

**How to cite this article**: Kim, J. A. *et al*. Presumed LRP1-targeting transport peptide delivers β-secretase inhibitor to neurons in vitro with limited efficiency. *Sci. Rep.* **6**, 34297; doi: 10.1038/srep34297 (2016).

## Supplementary Material

Supplementary Information

Supplementary video SV1

Supplementary video SV2

Supplementary video SV3

Supplementary video SV4

Supplementary video SV5

Supplementary video SV6
