# Supplementary material for: Presumed LRP1-targeting transport peptide delivers β-secretase inhibitor to neurons in vitro with limited efficiency
Source: Sci Rep. 2016 Sep 29;6:34297. doi: 10.1038/srep34297 (PMC5041153; doi:10.1038/srep34297)
Supplement: Supplementary Information [file srep34297-s1.pdf]

Supplementary Information

Presumed LRP1-targeting transport peptide delivers β-secretase inhibitor to neurons in vitro with limited efficiency

Jong-Ah Kim, Tommaso Casalini, Davide Brambilla & Jean-Christophe Leroux**

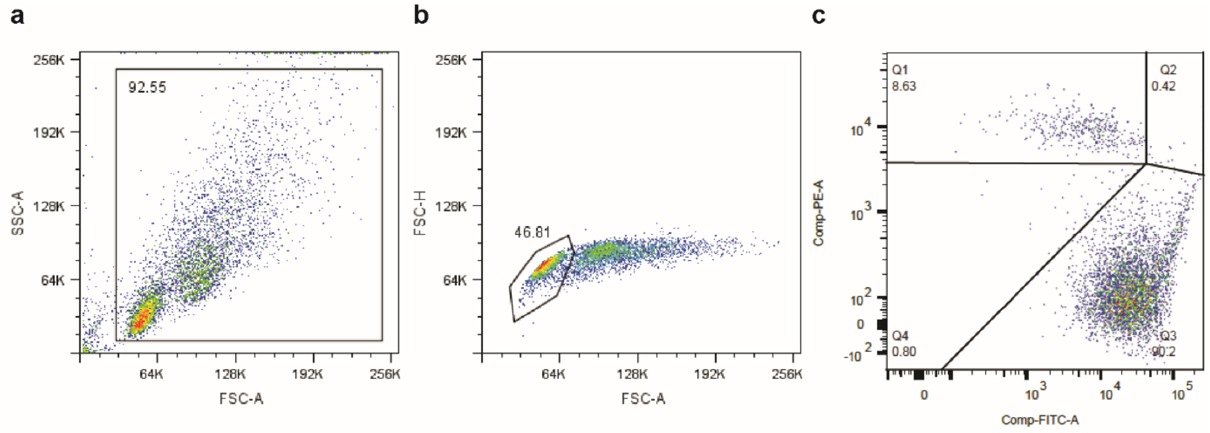

Figure S1 – Gating used in flow cytometry experiments. (a) Selection of the SH-SY5Y cell population cluster based on FSC/SSC and its separation from cell debris. **(b)** Selection of single cells from aggregates. **(c)** Viability dual staining in which viable cells are positively stained with calcein AM in green (FITC) and dead cells in red with ethidium homodimer-1 (PE). The plot shows that the single cells gate shown in (b) contained mostly viable cells (Q3=90.2%), while dead cells (Q1=8.6%) constituted a small fraction of the cluster analyzed.

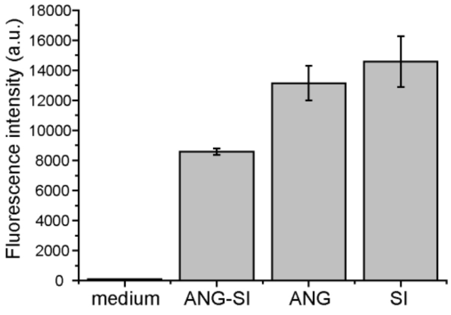

Figure S2 – Fluorescence intensity of TAMRA-labeled ANG-SI, ANG and SI peptides. Measurements were performed in FBS-supplemented medium. Mean ± SD (n=3).

a

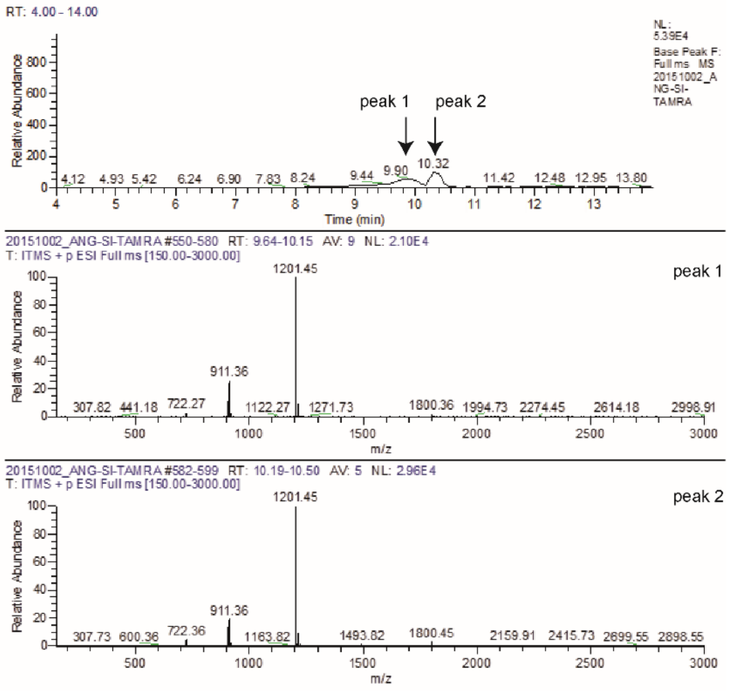
RT: 4.00 - 14.00
NL: 5.39E4
Base Peak F: Full ms MS 20151002_ANG-SI-TAMRA
Relative Abundance
peak 1
peak 2
4.12 4.93 5.42 6.24 6.90 7.83 8.24 9.44 9.90 10.32 11.42 12.48 12.95 13.80
Time (min)
20151002_ANG-SI-TAMRA #550-580 RT: 9.64-10.15 AV: 9 NL: 2.10E4
T: ITMS + p ESI Full ms [150.00-3000.00]
peak 1
1201.45
911.36
307.82 441.18 722.27 1122.27 1271.73 1800.36 1994.73 2274.45 2614.18 2998.91
m/z
20151002_ANG-SI-TAMRA #582-599 RT: 10.19-10.50 AV: 5 NL: 2.96E4
T: ITMS + p ESI Full ms [150.00-3000.00]
peak 2
1201.45
911.36
307.73 600.36 722.36 1163.82 1493.82 1800.45 2159.91 2415.73 2699.55 2898.55
m/z

b

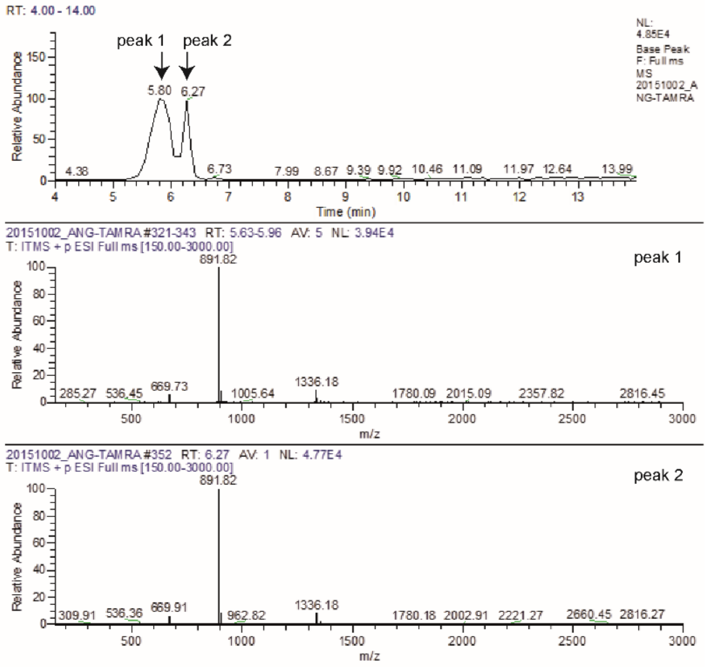
RT: 4.00 - 14.00
NL: 4.85E4
Base Peak F: Full ms MS 20151002_ANG-TAMRA
Relative Abundance
peak 1
peak 2
5.80 6.27
4.38 6.73 7.99 8.67 9.39 9.92 10.46 11.09 11.97 12.64 13.99
Time (min)
20151002_ANG-TAMRA #321-343 RT: 5.63-5.96 AV: 5 NL: 3.94E4
T: ITMS + p ESI Full ms [150.00-3000.00]
peak 1
891.82
285.27 536.45 669.73 1005.64 1336.18 1780.09 2015.09 2357.82 2816.45
m/z
20151002_ANG-TAMRA #352 RT: 6.27 AV: 1 NL: 4.77E4
T: ITMS + p ESI Full ms [150.00-3000.00]
peak 2
891.82
309.91 536.36 669.91 962.82 1336.18 1780.18 2002.91 2221.27 2660.45 2816.27
m/z

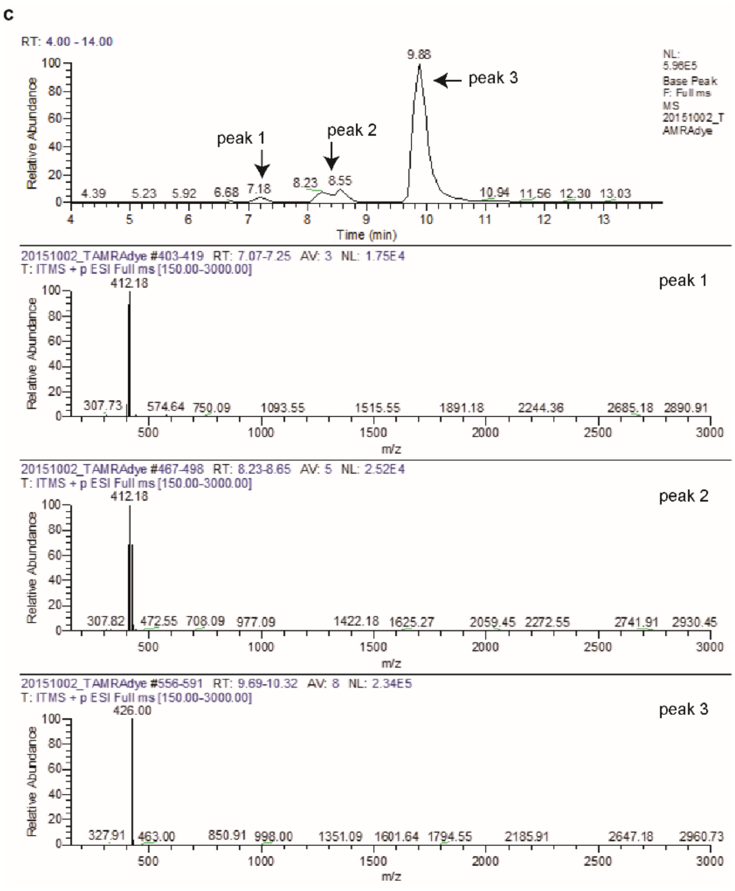

Figure S3 – Absence of unconjugated dye in the TAMRA-labeled peptides. The stocks of fluorescent peptides **(a)** ANG-SI and **(b)** ANG were analyzed by liquid chromatography–mass spectrometry (LC-MS) for their purity, molecular weight and presence of **(c)** free TAMRA dye.

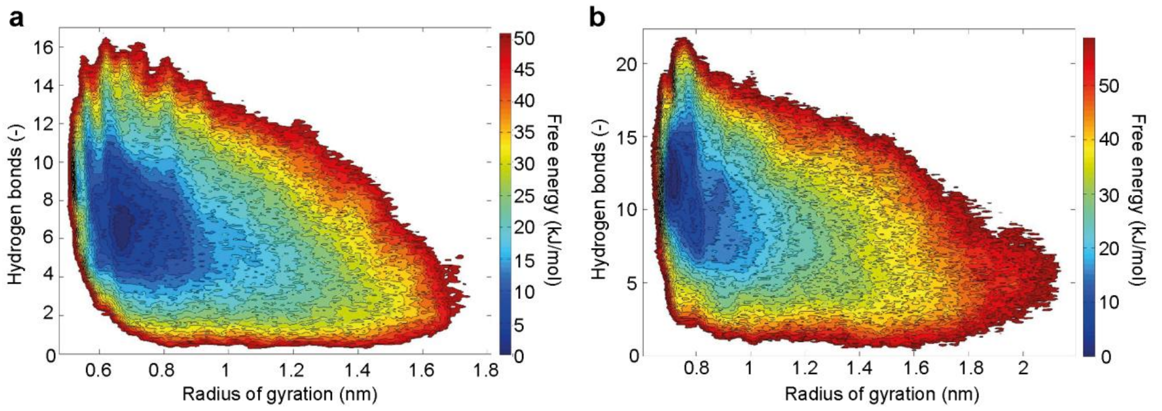

Figure S4 – Free energy surface for ANG and ANG-SI at 310 K as a function of radius of gyration of α-carbon atoms of protein backbone and intrapeptide hydrogen bonds. Metadynamics simulations allowed obtaining free energy surfaces for **(a)** ANG and **(b)** ANG-SI as a function of R_g and H_b, chosen as

relevant conformational parameters. Contour lines are plotted every k_BT unit (2.58 kJ mol^{-1}). Blue areas correspond to free energy minima, and thus are representative of the most probable folded structures in solution.

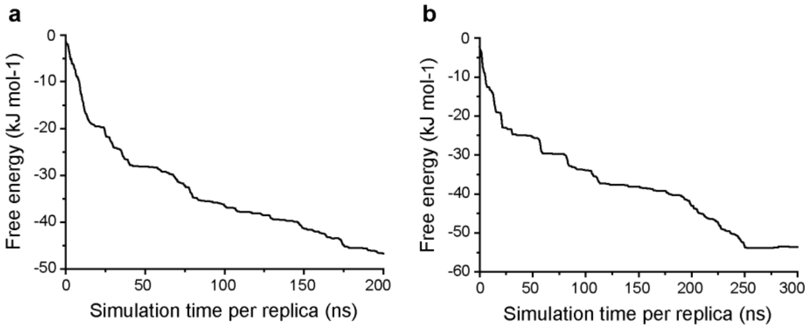

Figure S5 – Free energy for ANG and ANG-SI at 310 K as a function of simulation time. Folding free energy was computed as a function of time in order to check calculation convergence for **(a)** ANG and **(b)** ANG-SI systems. Convergence was considered to be reached when the free energy value reached a reasonable plateau with respect to the simulation time.

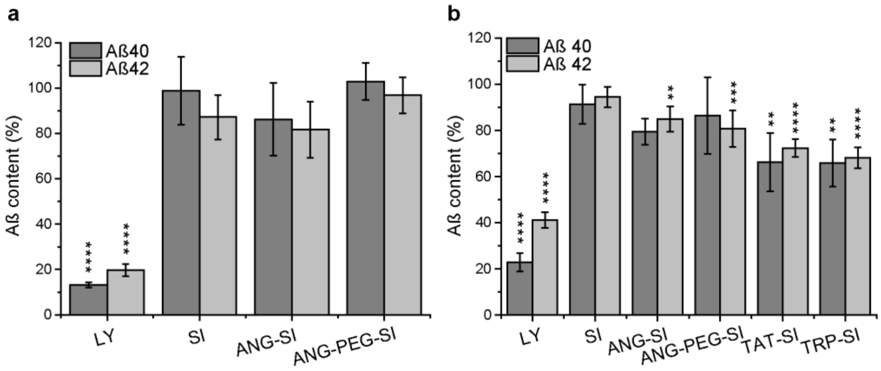

Figure S6– Activity of the bioconjugates on SH-SY5Y cells overexpressing the APP_{695} and APP_{751} isoforms. BACE1-inhibitory activity of LY2811376 (LY), SI, ANG-SI, ANG-PEG-SI, TAT-SI and TRP-SI after 6 h of continuous incubation at 5 μM in FBS-supplemented medium using SH-SY5Y cells overexpressing **a)** APP_{695} or **b)** APP_{751}.[1] Mean ± SD (n=3). **P<0.01, ***P<0.001 and ****P<0.0001, one-way ANOVA followed by Dunnet test.

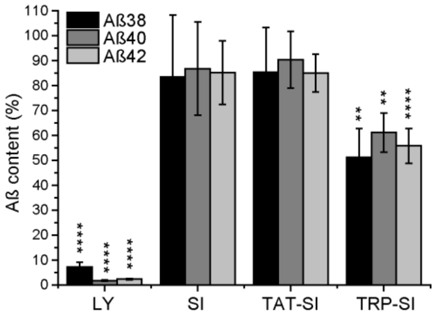

Figure S7 – Activity of the TAT-SI and TRP-SI bioconjugates on swAPP-overexpressing SH-SY5Y cells. BACE1-inhibitory activity of LY2811376 (LY), SI, TAT-SI and TRP-SI after 6 h of continuous incubation at 5 µM in FBS-supplemented medium. Mean ± SD (n=3). ***P*<0.01 and *****P*<0.0001, one-way ANOVA followed by Dunnet test.

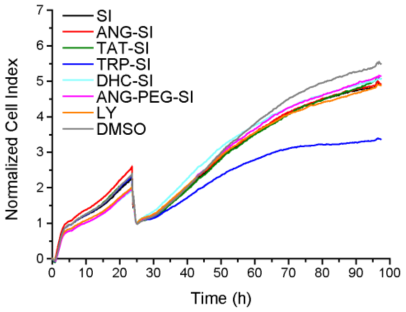

Figure S8 – Cytotoxicity of the peptides. Cell growth was monitored over 72 h using wild type SH-SY5Y cells during continuous exposure to the peptides at 5 µM using a Real-Time Cell Analysis (RTCA) xCELLigence system from Roche (Rotkreuz, Switzerland). Briefly, cells were seeded on the E-plates (10,000 cells/well) and after one day the medium was replaced by fresh medium containing the corresponding peptide and the cells were grown for 72 h. Cell growth was monitored with the RTCA system over 72 h during continuous exposure to the peptides at 5 µM.

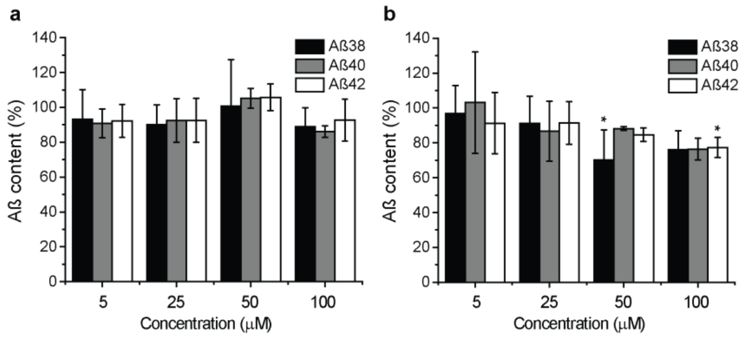

Figure S9 – Activity of ANG and SI at higher concentrations on swAPP-overexpressing SH-SY5Y cells. BACE1-inhibitory activity of a range of concentrations (5-100 µM) of **(a)** ANG and **(b)** SI after 6 h of continuous incubation in FBS-supplemented medium. Mean ± SD (n=3). **P*<0.05, one-way ANOVA followed by Dunnet test.

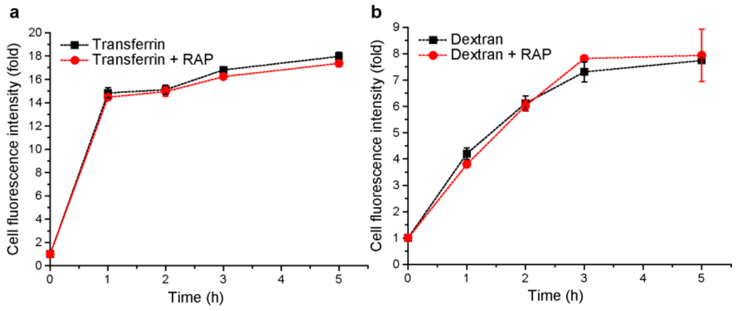

Figure S10 – Effect of RAP on LRP1-independent uptake. Mean cell fluorescence of wild type SH-SY5Y cells upon pre-incubation with RAP, followed by addition of **a)** fluorescently labeled human transferrin or **b)** FITC-labeled 70 kDa Dextran. Mean ± SD (n=3).

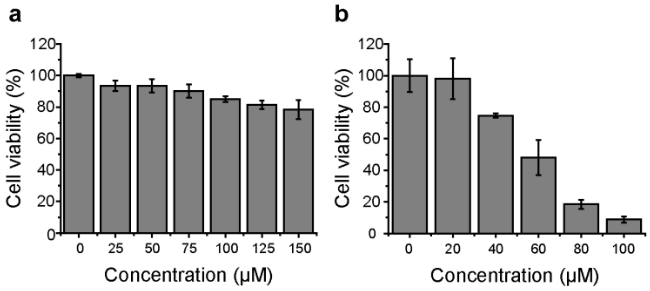

Figure S11 – Cytotoxicity induced by the endocytosis inhibitors. Cytotoxicity measured with the (3-(4,5-dimethylthiazol-2-yl)-5-(3-carboxymethoxyphenyl)-2-(4-sulfophenyl)-2H-tetrazolium) assay (MTS)

after exposure to increasing concentrations of **a)** Dynasore in serum-free medium or **b)** EIPA in serum-supplemented medium. Cell viability percentages are calculated taking vehicle-treated cells (medium with an equivalent percentage of DMSO) as 100%. Mean ± SD (n=3). Briefly, cells were seeded in 96-well plates at a density of approximately 30,000 cells/well and on the next day they were treated for 1.5 h with either Dynasore (25-150 μM) in serum-free medium or EIPA (20-100 μM) in serum-supplemented medium. The CellTiter 96 AQueous One Solution Cell Proliferation Assay from Promega (Madison, WI) was used following manufacturer's instructions. Upon treatment with Dynasore or EIPA the cells were incubated with the MTS reagent for 3 h at 37 °C after which the absorbance was measured at 490 nm with a plate reader. Cell-free solutions containing different concentrations of the compounds were measured as well and used to subtract background absorbance from the presented values.

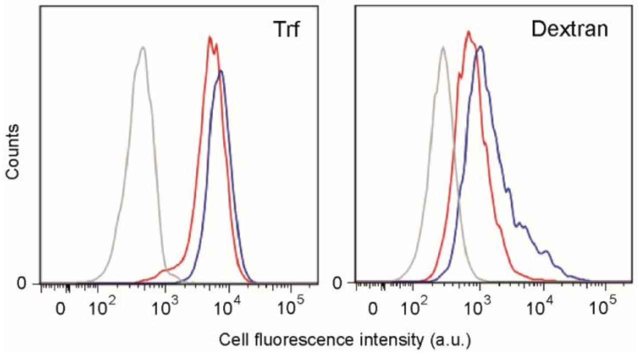

Figure S12 – Pharmacological inhibition of endocytosis. Cell fluorescence intensity distributions of cells treated with 100 μM Dynasore (left) or 60 μM EIPA (right). After 30min pre-treatment with the corresponding inhibitor (red) or medium alone (blue), cells were incubated for 1 h with 5 μg/mL fluorescently labeled human transferrin (Trf) or 0.75 mg/mL 70 kDa Dextran (Dextran) in presence (red) or absence (blue) of the same inhibitor used in the pre-treatment. Background cell fluorescence of only inhibitor-treated cells is shown in grey. Distributions shown are representative examples of at least three independent experiments performed in triplicates.

Metadynamics simulations

Metadynamics simulations allow to reconstruct the free energy of the system by keeping track of the sampling of conformations and discouraging the sampling of structures already visited during the simulations, as a function of few conformational degrees of freedom (collective variables).[2,3] Calculations were performed adopting the approach discussed by Deighan *et al.*, who combined metadynamics and parallel tempering (PTMetaD) in the well-tempered ensemble (PTMetaD-WTE) in order to improve simulation efficiency.[4] All simulations were performed with GROMACS 5.0.2[5] patched with PLUMED 2.1.0 plugin[6]. Linear ANG and ANG-SI structures were built using *tleap* module implemented in *AmberTools* package. Each peptide was solvated with about 14500 TIP3P water molecules;[7] Na_+ and Cl_- ions (whose parameters were taken from Joung and Cheatham[8]) were added in order to assure electroneutrality and to mimic PBS environment. The ff14SB force field parameters were employed for ANG and ANG-SI; since statin is not a standard residue in ff14SB libraries, atomic charges were derived with a proper protocol (*vide infra*).[9] The starting system for PTMetaD-WTE simulations was obtained according to the following protocol. First of all, energy minimization was carried out in order to remove bad solvent/solute and solvent/solvent contacts. Then temperature was raised from 0 to 310 K through 200 ps in NVT ensemble, applying a weak harmonic restraint on the solute in order to avoid wild fluctuations. Finally, the system was equilibrated through 1 ns simulation in NPT ensemble at 310 and 1 atm. Velocity rescale algorithm and Parrinello-Rahman barostat[10,11] were used in order to maintain temperature and pressure at the desired values. Electrostatic interactions were computed by means of Particle Mesh Ewald (PME) method using a cutoff value equal to 1.2 nm; the same cutoff was employed for Van Der Waals interactions.[12] Neighbor list was updated every 5 fs; all covalent bonds involving hydrogen were restrained using LINCS algorithm.[13] PTMetaD-WTE simulations were performed using 12 replicas, whose temperature ranges from 310 to 588 K; before starting the simulations, 1 ns molecular dynamics simulation were carried out for each replica, so that every system could equilibrate according to its temperature and adjacent replicas exhibit different peptide conformations. A two-step protocol was used for PTMetaD-WTE simulations. First of all, a 1 ns PTMetaD simulation was performed biasing only potential energy, chosen as collective

variable.[14] Gaussian were deposited every 0.5 ps, with an height value equal to 0.28 kcal mol^{-1}, a width value equal to 956 kcal mol^{-1} and a bias factor equal to 24. In the following step, the radius of gyration related to α-carbon atoms in the protein backbone (R_g) and the intrapeptide hydrogen bonds (H_b) were chosen as collective variables, since they proved to be suitable for the sampling of protein structures in water solutions,[4,15-17] and biased using the well-tempered metadynamics algorithm.[3] No more Gaussians were added in order to bias potential energy, since the cumulative bias was employed as a static additional bias. For what regards radius of gyration and intrachain hydrogen bonds, Gaussians were added every 1 ps, using a height value equal to 0.28 kcal mol^{-1}, width values equal to 0.01 and 0.1 for R_g and H_b respectively and a bias factor equal to 8. Temperature control and long-range interactions were treated as described above. Simulation times equal to 200 ns and 300 ns were used for each replica for ANG and ANG-SI respectively, leading to a total simulation time of 2.4 μs for ANG and 3.6 μs for ANG-SI. Exchange attempts between replicas were carried out every 0.4 ps by means of PTMetaD algorithm implemented in GROMACS 5.0.2 patched with PLUMED 2.1.0 plugin. The convergence of the simulations was checked by evaluating the folding free energy as a function of simulation time; details are reported in Figure S9a and b. A typical output of metadynamics simulations is a free energy surface as a function of the chosen collective variables; free energy surfaces for ANG and ANG-SI as a function of R_g and H_b are shown in Figures S10 a and b.

Molecular dynamics simulations

Molecular dynamics simulations were performed adopting the same protocol described above for obtaining the starting system for metadynamics simulations. Folded ANG and ANG-SI were solvated with about 14500 TIP3P water molecules; Na+ and Cl- ions were added in order to assure electroneutrality and mimic PBS environment. After energy minimization, temperature was raised from 0 to 300 K in NVT ensemble. The system was then equilibrated for 1 ns in NPT ensemble in order to reach the correct density, and then 50 ns molecular dynamics simulations were performed at 310 K and 1 atm. Temperature and pressure were kept at the desired values by means of velocity rescaling algorithm and Parrinello-Rahman barostat respectively. Long-

range interactions were treated as described. The same force fields employed for metadynamics simulations were employed.

Statin atomic charges

Statin atomic charges were derived adopting a calculation protocol consistent with force field design.[9,18] The initial statin structure was taken from crystallographic structure 3LIY in pdb database; the molecule of interest is here included in a small peptide, and thus constitutes a consistent initial guess for charge derivation. Statin was capped with acetyl (ACE) and N-methyl (NME) groups; the obtained structure was firstly minimized through *ab initio* calculations at MP2/6-31G* level of theory. Electrostatic potentials were then computed from the optimized structure at HF/6-31G* level of theory. Atomic charges were then fitted adopting RESP formalism starting from electrostatic potentials.[19] A two-step protocol was adopted: first, atomic charges were fitted assigning an overall charge for statin equal to 0, while ACE and NME atomic charges were taken from force field library. In the second step, charge equivalence for chemically equivalent atoms was imposed.

Trajectories post-processing

Electrostatic potentials were computed with Adaptive Poisson Boltzmann Solver (APBS).[20] Electrostatic potential is expressed in k_BTe^{-1} units, where k_B is Boltzmann constant, T is the absolute temperature and e is the electron charge. Average solvation free energy was computed through MMPBSA as implemented in *AmberTools* package.[21]

References

1. Belyaev, ND, Kellett, KAB, Beckett, C, Makova, NZ, Revett, TJ, Nalivaeva, NN *et al.* (2010). The transcriptionally active amyloid precursor protein (app) intracellular domain is preferentially produced from the 695 isoform of app in a β-secretase-dependent pathway. *J Biol Chem* **285**: 41443-41454.
2. Laio, A & Parrinello, M. (2002). Escaping free-energy minima. *Proceedings of the National Academy of Sciences of the United States of America* **99**: 12562-12566.
3. Barducci, A, Bussi, G & Parrinello, M. (2008). Well-tempered metadynamics: A smoothly converging and tunable free-energy method. *Phys Rev Lett* **100**.
4. Deighan, M, Bonomi, M & Pfaendtner, J. (2012). Efficient simulation of explicitly solvated proteins in the well-tempered ensemble. *Journal of Chemical Theory and Computation* **8**: 2189-2192.

5. Pronk, S, Pall, S, Schulz, R, Larsson, P, Bjelkmar, P, Apostolov, R *et al.* (2013). Gromacs 4.5: A high-throughput and highly parallel open source molecular simulation toolkit. *Bioinformatics* **29**: 845-854.
6. Tribello, GA, Bonomi, M, Branduardi, D, Camilloni, C & Bussi, G. (2014). Plumed 2: New feathers for an old bird. *Comput Phys Commun* **185**: 604-613.
7. Jorgensen, WL, Chandrasekhar, J, Madura, JD, Impey, RW & Klein, ML. (1983). Comparison of simple potential functions for simulating liquid water *J Chem Phys* **79**: 926 - 936.
8. Joung, IS & Cheatham, TE. (2008). Determination of alkali and halide monovalent ion parameters for use in explicitly solvated biomolecular simulations. *J Phys Chem B* **112**: 9020-9041.
9. Maier, JA, Martinez, C, Kasavajhala, K, Wickstrom, L, Hauser, KE & Simmerling, C. (2015). Ff14sb: Improving the accuracy of protein side chain and backbone parameters from ff99sb. *Journal of Chemical Theory and Computation* **11**: 3696-3713.
10. Bussi, G, Donadio, D & Parrinello, M. (2007). Canonical sampling through velocity rescaling. *J Chem Phys* **126**.
11. Parrinello, M & Rahman, A. (1981). Polymorphic transitions in single-crystals - a new molecular-dynamics method. *J Appl Phys* **52**: 7182-7190.
12. Darden, T, York, D & Pedersen, L. (1993). Particle mesh ewald - an n.Log(n) method for ewald sums in large systems. *J Chem Phys* **98**: 10089-10092.
13. Hess, B, Bekker, H, Berendsen, HJC & Fraaije, JGEM. (1997). Lincs: A linear constraint solver for molecular simulations. *J Comput Chem* **18**: 1463-1472.
14. Bussi, G, Gervasio, FL, Laio, A & Parrinello, M. (2006). Free-energy landscape for beta hairpin folding from combined parallel tempering and metadynamics. *J Am Chem Soc* **128**: 13435-13441.
15. Barducci, A, Bonomi, M, Prakash, MK & Parrinello, M. (2013). Free-energy landscape of protein oligomerization from atomistic simulations. *Proceedings of the National Academy of Sciences of the United States of America* **110**: E4708-E4713.
16. Berteotti, A, Barducci, A & Parrinello, M. (2011). Effect of urea on the beta-hairpin conformational ensemble and protein denaturation mechanism. *J Am Chem Soc* **133**: 17200-17206.
17. Owczarz, M, Casalini, T, Motta, AC, Morbidelli, M & Arosio, P. (2015). Contribution of electrostatics in the fibril stability of a model ionic-complementary peptide. *Biomacromolecules* **16**: 3792-3801.
18. Cornell, WD, Cieplak, P, Bayly, CI, Gould, IR, Merz, KM, Ferguson, DM *et al.* (1996). A second generation force field for the simulation of proteins, nucleic acids, and organic molecules (vol 117, pg 5179, 1995). *J Am Chem Soc* **118**: 2309-2309.
19. Bayly, CI, Cieplak, P, Cornell, WD & Kollman, PA. (1993). A well-behaved electrostatic potential based method using charge restraints for deriving atomic charges - the resp model. *J Phys Chem* **97**: 10269-10280.
20. Baker, NA, Sept, D, Joseph, S, Holst, MJ & McCammon, JA. (2001). Electrostatics of nanosystems: Application to microtubules and the ribosome. *Proceedings of the National Academy of Sciences of the United States of America* **98**: 10037-10041.
21. Miller, BR, McGee, TD, Swails, JM, Homeyer, N, Gohlke, H & Roitberg, AE. (2012). Mmpbsa.Py: An efficient program for end-state free energy calculations. *Journal of Chemical Theory and Computation* **8**: 3314-3321.

Supplementary videos

Video SV1 – Time-lapse video corresponding to the live-cell confocal microscopy images shown in Fig 4a. Wild-type SH-SY5Y cells transfected to express the GFP-labeled version of Rab7a, a marker of late endosomes (green, LE-GFP), were incubated for 1 h with 3 μM fluorescently labeled ANG-SI conjugate (red) after which they were washed and imaged live every 5 sec for 2 min at 37 °C and 5% CO_2.

Video SV2 – Time-lapse video of live-cell confocal microscopy of ANG-SI and LE-GFP. Wild-type SH-SY5Y cells transfected to express the GFP-labeled version of Rab7a, a marker of late endosomes (green, LE-GFP), were incubated for 1 h with 3 μM fluorescently labeled ANG-SI conjugate (red) after which they were washed and imaged live every 5 sec for 2 min at 37 °C and 5% CO_2.

Video SV3 – Time-lapse video of live-cell confocal microscopy of ANG-SI and LE-GFP. Wild-type SH-SY5Y cells transfected to express the GFP-labeled version of Rab7a, a marker of late endosomes (green, LE-GFP), were incubated for 1 h with 3 μM fluorescently labeled ANG-SI conjugate (red) after which they were washed and imaged live every 5 sec for 2 min at 37 °C and 5% CO_2.

Video SV4 – Time-lapse video corresponding to the live-cell confocal microscopy images shown in Fig 4b. Wild-type SH-SY5Y cells transfected to express the GFP-labeled version of BACE1 (green, BACE1-GFP), were incubated for 1 h with 3 μM fluorescently labeled ANG-SI conjugate (red) after which they were washed and imaged live every 5 sec for 2 min at 37 °C and 5% CO_2.

Video SV5 – Time-lapse video of live-cell confocal microscopy of ANG-SI and BACE1-GFP. Wild-type SH-SY5Y cells transfected to express the GFP-labeled version of BACE1 (green, BACE1-GFP), were incubated for 1 h with 3 μM fluorescently labeled ANG-SI conjugate (red) after which they were washed and imaged live every 5 sec for 2 min at 37 °C and 5% CO_2.

Video SV6 – Time-lapse video of live-cell confocal microscopy of ANG-SI and BACE1-GFP. Wild-type SH-SY5Y cells transfected to express the GFP-labeled version of BACE1 (green, BACE1-GFP), were incubated for 1 h with 3 μM fluorescently labeled ANG-SI conjugate (red) after which they were washed and imaged live every 5 sec for 2 min at 37 °C and 5% CO_2.
