# Supplementary figures and images for: Presumed LRP1-targeting transport peptide delivers β-secretase inhibitor to neurons in vitro with limited efficiency
Source: Sci Rep. 2016 Sep 29;6:34297. doi: 10.1038/srep34297 (PMC5041153; doi:10.1038/srep34297)

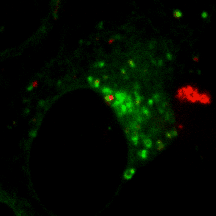

Supplement: Supplementary video SV1 [file srep34297-s2.gif]

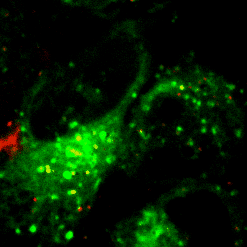

Supplement: Supplementary video SV2 [file srep34297-s3.gif]

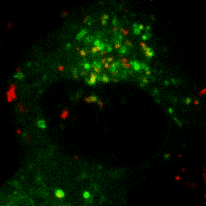

Supplement: Supplementary video SV3 [file srep34297-s4.gif]

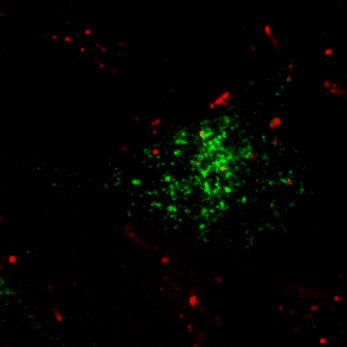

Supplement: Supplementary video SV4 [file srep34297-s5.gif]

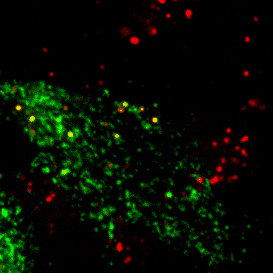

Supplement: Supplementary video SV5 [file srep34297-s6.gif]

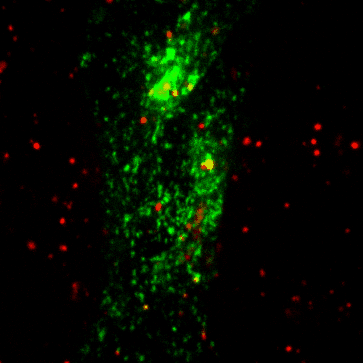

Supplement: Supplementary video SV6 [file srep34297-s7.gif]
